# Dual Crosslinked Antioxidant Mixture of Poly(vinyl alcohol) and Cerium Oxide Nanoparticles as a Bioink for 3D Bioprinting

**DOI:** 10.1021/acsanm.3c02962

**Published:** 2023-09-25

**Authors:** Nasera Rizwana, Namrata Maslekar, Kaushik Chatterjee, Yin Yao, Vipul Agarwal, Manasa Nune

**Affiliations:** † Manipal Institute of Regenerative Medicine (MIRM), Bengaluru, Manipal Academy of Higher Education (MAHE), Manipal 576104, Karnataka, India; ‡ Cluster for Advanced Macromolecular Design (CAMD), School of Chemical Engineering, University of New South Wales, Sydney, New South Wales 2052, Australia; § Department of Materials Engineering, Indian Institute of Science, Bangalore 560012, India; ∥ Electron Microscope Unit, Mark Wainwright Analytical Centre, University of New South Wales, Sydney, New South Wales 2052, Australia

**Keywords:** bioink, antioxidant, 3D bioprinting, nanoceria, dual crosslinking, poly(vinyl alcohol)

## Abstract

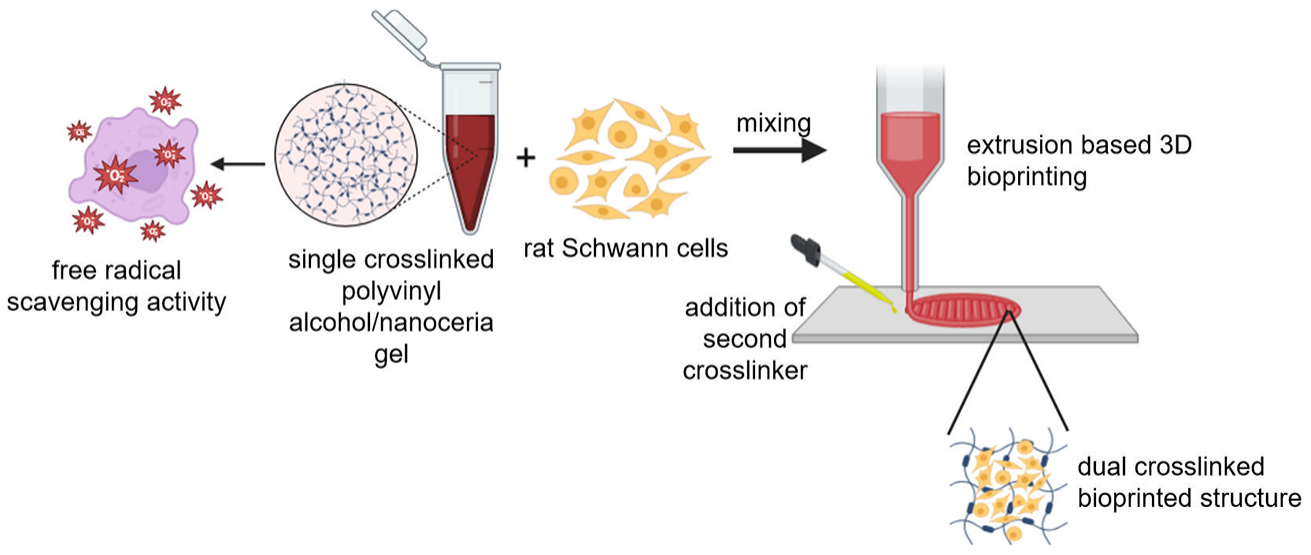

Three-dimensional (3D) bioprinting has made it possible to fabricate structures with intricate morphologies and architectures, which is considered difficult to do when using other conventional techniques like electrospinning. Although the 3D printing of thermoplastics has seen a huge boom in the past few years, it has been challenging to translate this technology to cell-based printing. A major limitation in bioprinting is the lack of inks that allow for the printing of 3D structures that meet the biological requirements of a specific organ or tissue. A bioink is a viscous polymer solution that cells are incorporated into before printing. Therefore, a bioink must have specific characteristics to ensure both good printability and biocompatibility. Despite the progress that has been made in bioprinting, achieving a balance between these two properties has been difficult. In this work, we developed a multimodal bioink that serves as both a cell carrier and a free radical scavenger for treating peripheral nerve injury. This bioink comprises poly(vinyl alcohol) (PVA) and cerium oxide nanoparticles (also called nanoceria (NC)) and was developed with a dual crosslinking method that utilizes citric acid and sodium hydroxide. By employing this dual crosslinking method, good printability of the bioink and shape fidelity of the bioprinted structure were achieved. Additionally, a cell viability study demonstrated that the cells remained compatible and viable even after they underwent the printing process. The combination of this PVA/NC bioink and the dual crosslinking method proved to be effective in enhancing printability and cell biocompatibility for extrusion-based bioprinting applications.

## 1 Introduction

In recent years, significant advancements have been made in the field of three-dimensional (3D) bioprinting, enabling the fabrication of intricate, 3D, functional tissue constructs by printing compatible biomaterials and viable cells.^1−4^ This progress has created new opportunities for certain applications, such as organ transplantations and drug screenings. Although simple organs have been successfully bioprinted, more complex organs like nerves present challenges due to their intricate architectures and complex anatomical features.^5^ To address the growing demand for organ transplants, tissue engineering has emerged as a promising alternative solution.

The integration of 3D printing technology in tissue engineering has revolutionized the creation of functional and implantable tissue constructs. Additive manufacturing techniques are employed to fabricate biomaterial scaffolds with patient-specific geometries and porous networks that facilitate tissue growth.^6,7^ Traditional methods involve fabricating the scaffolds first and then incorporating the cells, which can result in a non-uniform cell distribution.^8−11^ However, the emerging field of bioprinting allows for the simultaneous printing of cells and biomaterials, enabling precise cell placement within the printed tissues.^12^ Bioprinting techniques can be categorized as vat polymerization, material extrusion, or material jetting.^13−15^ Vat polymerization-based printing involves the fabrication of scaffolds, onto which cell seeding is usually carried out. Traditionally, vat polymerization-based printing methods include stereolithography, digital light processing, and two-photon polymerization. Among these, stereolithography remains the most widely explored method, as it offers rapid printing at a speed of approximately 40,000 mm/s with high accuracy. However, one of the biggest limitations of vat polymerization-based printing methods is the dependence on light-sensitive materials.^15,16^ To mitigate this limitation, material jetting techniques have been developed, such as inkjet printing and laser-assisted printing. These techniques, which are characterized by high-speed printing with enhanced accuracy, resolution, and cost-effectiveness, overcome the material limitations of the vat polymerization techniques (i.e., the reliance on light-sensitive materials). However, material jetting techniques can be prone to nozzle clogging during printing.^14,17^ To overcome this challenge, extrusion-based printing has been developed. Extrusion-based printing is one of the most widely used bioprinting techniques. In a typical extrusion process, a bioink is loaded into cartridges, and pneumatic pressure is used to eject the biomaterial onto the printing platform. Extrusion-based printing has several advantages, including the ability to print with a high density of cells, the ability to obtain a uniform distribution of cells within the printed construct, and low costs when compared to other bioprinting approaches.^13,18−20^

Bioinks, which serve as the cell-delivery media in bioprinting, often consist of naturally derived proteins and polysaccharides, providing a cell-friendly environment resembling the natural extracellular matrix. An ideal bioink for bioprinting should possess specific mechanical, rheological, and chemical properties, as well as biological characteristics, that facilitate the printing process and ensure the fidelity of the desired shape.^21,22^ Biocompatibility and biodegradability are crucial factors to consider when developing a bioprinted scaffold for tissue regeneration. Various natural and synthetic biomaterials, including decellularized matrix components, hydrogels, microcarriers, cell aggregates, and stem cells, have been employed as bioinks in bioprinting research.^23−26^ Bioinks must exhibit biocompatibility and mimic the biochemical and physical environments of the targeted tissue. They must also exhibit appropriate printability to enable precise layer-by-layer construction and the ability to withstand mechanical stress. Achieving a balance between biocompatibility and printability poses a challenge, as higher viscosity can improve biocompatibility but may compromise printability. Higher-viscosity hydrogels pose three major drawbacks. First, cell sedimentation can occur within highly viscous fluid, causing a non-homogenous distribution of the cells within the hydrogel.^27−30^ Second, higher-viscosity hydrogels require a larger amount of shear forces, which can lead to cell death. And third, higher viscosities cause print heads to clog, which leads to improper printing.^31−33^ Therefore, the careful selection of the hydrogel and its crosslinking mechanism is becoming more important for overcoming the aforementioned disadvantages and obtaining shape fidelity and good cell viability post-bioprinting.

Poly(vinyl alcohol) (PVA) is a synthetic polymer that consists of hydroxyl functional groups and a carbon chain backbone. It has been explored in biomedical applications due to its hydrophilicity, biocompatibility, and biodegradability. It is a water-soluble polymer traditionally known to form bioscaffolds with low mechanical strength, which has been circumvented using crosslinking to enhance the overall physical properties. Crosslinking restricts the mobility of polymeric chains, thus affecting the chemical structure and crystallinity of the polymer. Furthermore, in the case of PVA, crosslinking has been shown to enhance cell adhesion by forming hydrogen bonds between the polar groups that exist on the cell surface and the hydroxyl groups of the polymer.^34−41^ One advantage of using PVA is the possibility of partial crosslinking by utilizing a portion of the hydroxyl groups present on the polymer backbone, thereby enabling further modification of the remaining hydroxyl groups. These remaining groups can then be utilized to introduce additional functionalities.^38,40^

Nanoceria (NC), also known as cerium oxide nanoparticles, is a unique rare earth metal oxide that can exist in both the trivalent state and the tetravalent state. It possesses a crystalline lattice structure, which makes its surface highly reactive for free radical neutralization. Because of its nanometer size, oxygen vacancies are formed in its lattice, creating oxygen defects for free radical scavenging.^42−44^ Free radicals play a crucial role in peripheral nerve injury. Excessive free radical production causes impaired DNA synthesis and an impaired mitochondrial structure in Schwann cells. Hence, it is necessary to inhibit free radical production to maintain the functionality of Schwann cells and their interactions with other types of cells following peripheral nerve injury. Furthermore, it should be noted that NC-containing inks have not yet been tested for bioprinting applications for treating peripheral nerve injury.^45−47^

In this work, we developed a 3D bioprinted nanocomposite scaffold by combining a hydrogel, nanoparticles, and a crosslinking method to prepare an appropriate bioink that could provide antioxidant properties and act as a cell carrier for treating peripheral nerve injury. We established the use of a combination of NC and poly(vinyl alcohol) (PVA), along with a dual crosslinking method, to prepare a bioink consisting of rat Schwann RSC96 cells. We optimized the bioink through a systematic investigation of the effects of varying the NC concentration and the dual crosslinking method. The PVA/NC hydrogel was first crosslinked with citric acid in order to obtain an adequate viscosity for bioprinting and to maintain good cell viability. Then, to provide improved shape fidelity to the printed structure, secondary crosslinking with sodium hydroxide was carried out after bioprinting was complete. This multifunctional PVA/NC nanocomposite scaffold was characterized regarding its free radical scavenging effectiveness, bioprinting properties, and the cell viability of the embedded cells.

## 2 Materials and Methods

### 2.1 Materials

Cerium(IV) oxide (a nanopowder with a particle size of <25 nm) was procured from Sigma-Aldrich, and poly(vinyl alcohol) (25–32 cPs) was purchased from Nice Chemicals. Citric acid anhydrous was purchased from Qualigens. Sodium hydroxide pellets, dimethyl sulfoxide (DMSO), and a 3-(3,4-dimethylthiazol-2-yl)-2,5-diphenyltetrazoliium bromide (MTT) assay (MB186) were purchased from HiMedia Laboratories. Rat Schwann cells (RSC96) were purchased from ATCC. Dulbecco’s modified eagle medium (DMEM), fetal bovine serum (FBS), penicillin-streptomycin, antibiotic-antimycotic, and 0.25% trypsin-EDTA were purchased from Gibco. 2,2-Diphenyl-1-picrylhydrazyl (DPPH) was purchased from SRL Chemical. 2′,7′-Dichlorodihydrofluorescein diacetate (H_2_DCFDA) and a live/dead viability/cytotoxicity kit were purchased from Thermo Fisher Scientific. The INKREDIBLE+ 3D bioprinter was purchased from CELLINK. Bioprinting nozzles were purchased from Alfatek Systems.

### 2.2 Fabrication of Poly(vinyl alcohol) (PVA) Gel and Nanoceria-Loaded PVA (PVA/NC) Gel and the Dual Crosslinking Strategy

The PVA gel was prepared by adding 25 wt % PVA to deionized water, which was then heated at 120 °C for 1 h. After the complete dissolution of PVA, the temperature was reduced to 40 °C and 5 wt % citric acid was added. The mixture was stirred for 10 min to induce primary crosslinking, which led to an increase in the viscosity to such an extent as to allow for printing using a bioprinter.

**Figure 1:**
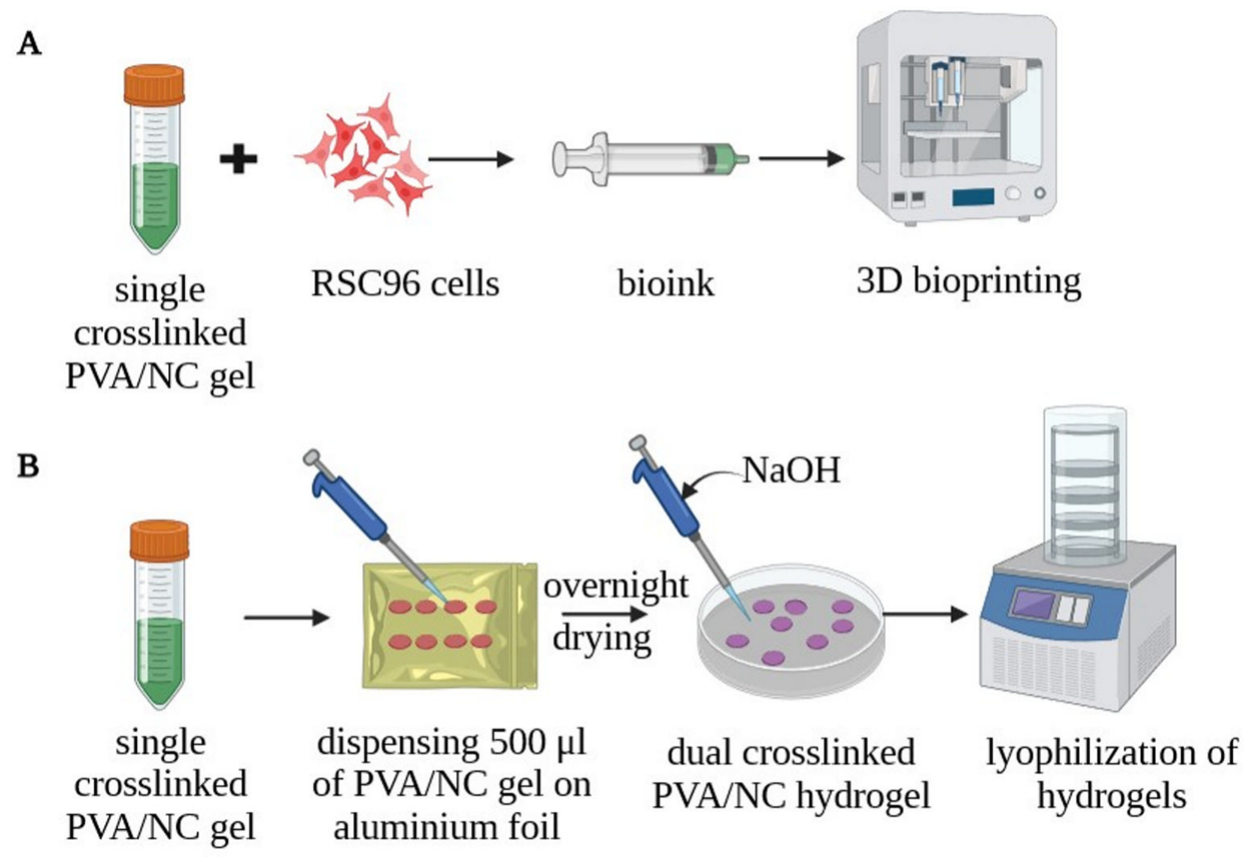
Schematics representing the methodology of (A) the preparation of the single crosslinked PVA/NC bioink and (B) the fabrication of the lyophilized PVA/NC hydrogels for characterization.

To prepare the PVA/NC gel, different concentrations (0.5%, 0.75%, 1%, and 2%) of nanoceria were dispersed in deionized water by ultrasonication in a water bath. This was followed by the addition of 25 wt % PVA to the NC dispersion, and the mixture was heated at 120 °C for 1 h. Subsequently, the temperature of the mixture was reduced to 40 °C and 5 wt % citric acid was added (acting as a primary crosslinker); the mixture was then stirred for 10 min. This led to the formation of citric-acid-crosslinked PVA/NC gels (which should not be confused with hydrogels) (Figure 1A).

To fabricate the PVA and PVA/NC standalone hydrogels, 500 μL of the citric-acid-crosslinked neat PVA and PVA/NC gels were placed over aluminum foil. The gels were allowed to dry overnight at room temperature. After drying, the gels were peeled off from the aluminum foil and placed in a 1% w/v 0.1 M NaOH aqueous solution for 2 min for secondary crosslinking to occur in order to obtain the hydrogels. After secondary crosslinking, the neat PVA and PVA/NC hydrogels were washed thrice with distilled water and dried at room temperature. To avoid moisture absorption, we placed the hydrogels in a desiccator before characterization. The hydrogels were lyophilized using an Alpha 2-4 LDplus lyophilizer before further analysis (Figure 1B).

### 2.3 Characterization of NC Particles and PVA/NC Hydrogels

#### 2.3.1 Scanning Electron Microscopy (SEM) and Energy Dispersive X-ray Spectroscopy (EDS)

Scanning electron microscopy was performed on the NC particles and lyophilized PVA/NC hydrogels to analyze their surface morphologies using an FEI Nova NanoSEM 230 FE-SEM microscope. Elemental analysis was performed using the energy dispersive X-ray spectrometer (EDS) on the SEM. A small amount of the NC powder and the lyophilized hydrogels were placed on adhesive double-sided carbon tape and sputter-coated with gold prior to imaging.

#### 2.3.2 X-ray Diffraction (XRD)

XRD was used to characterize the NC particles and lyophilized PVA/NC hydrogels. All of the samples were scanned at a diffraction angle 2θ ranging from 10 to 90° at a rate of 2°/min using Cu Kα_1_ radiation on a Malvern PANalytical Empyrean II X-ray diffractometer, operating at 40 kV and using a 40 mA current.

#### 2.3.3 Fourier Transform Infrared (FTIR) Spectroscopy

FTIR spectroscopy was performed on the NC particles and lyophilized PVA/NC hydrogels using a Bruker IFS66/S instrument. The measurements were carried out from 400 to 4000 cm^–1^ at a wavenumber resolution of 4 cm^–1^, and 16 scans per sample are averaged and presented.

#### 2.3.4 Dynamic Light Scattering (DLS) and Zeta Potential Analyses

Dynamic light scattering and zeta potential analyses of the NC particles were carried out using a Malvern Zetasizer Nano Series instrument. Samples were dispersed in Milli-Q water, and the particle size was recorded in triplicate with an average of at least 11 measurements per replicate. Zeta potential measurements were conducted in triplicate. Both the particle size and zeta potential data are reported as averages of the three replicates.

#### 2.3.5 Nanoceria Release Study

NC release from the lyophilized hydrogels was conducted in phosphate-buffered saline (PBS). The absorbance of the PBS containing the lyophilized hydrogels (200 μL) was taken at different time points using a multiplate reader (HH34000000, EnSight, PerkinElmer). The concentration of released NC from the lyophilized PVA/NC hydrogels was calculated by using the NC standard curve. To obtain the calibration curve, a nanoceria solution of 400 μg/mL was serially diluted to make 200, 100, and 50 μg/mL solutions, and the absorbance of the dispersed solutions was measured at 305 nm using a multiplate reader. A linear correlation was determined for the calibration curve, and a regression coefficient of 0.9897 was obtained. The cumulative concentration of the nanoceria released from the hydrogels was calculated.

#### 2.3.6 Free Radical Scavenging Activity (DPPH) Assay

The free radical scavenging activity of lyophilized neat PVA and all concentrations of the lyophilized PVA/NC hydrogels was assessed using a 2,2-diphenyl-1-picrylhydrazyl (DPPH) assay.^47^ The lyophilized hydrogels of equal weights were placed in a PBS solution for 2 days to allow them to swell. Then, the swelled hydrogels were placed in 1 mL of 100% ethanol for 24 h. Simultaneously, a 0.1 mM DPPH solution in ethanol was prepared separately. Next, 4 mL of this DPPH ethanol solution was added to 1 mL of the 24 h hydrogel-treated ethanol, and the mixture was stirred vigorously for 1 min. The DPPH-mixed solution was then incubated in the dark for 20 min to allow the reaction to occur. A DPPH blank was used as a control. The absorbance of the solution comprising DPPH and the ethanol-treated samples was quantified at a wavelength of 517 nm using a multiplate reader (HH34000000, EnSight, PerkinElmer). The radical scavenging activity was calculated using eq 1:
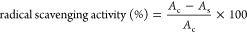
1where *A*_c_ is the absorbance of the control and *A*_s_ is the absorbance of the hydrogel-treated ethanol.

#### 2.3.7 Free Radical Scavenging Activity (H_2_DCFDA) Assay

The radical scavenging activity of the lyophilized PVA/NC hydrogels was further evaluated by completing a 2′,7′-dichlorodihydrofluorescein diacetate (H_2_DCFDA) assay following the previously described procedure.^48,49^ In brief, the lyophilized hydrogels were placed in DMEM medium without FBS at 37 °C in a humidified incubator for 1 day and 7 days to allow for NC release. After days 1 and 7, the NC-released media (termed conditioned media) was collected and used directly for this assay. Rat Schwann RSC96 cells (5 × 10^4^ cells/well) were seeded in a 24-well plate and allowed to adhere for 24 h in a humidified incubator with 5% CO_2_ at 37 °C. Then, the cells were treated with 300 μM H_2_O_2_ for 30 min at 37 °C to allow for the generation of reactive oxygen species (ROS). Next, the cells were washed with PBS and treated with the conditioned media for 24 h in an incubator with 5% CO_2_ at 37 °C. Afterward, the conditioned media was removed and the cells were stained with 200 μL of a 100 μM H_2_DCFDA solution in ethanol and incubated in an incubator at 37 °C for 45 min in the dark. The cells were then washed with PBS, and fluorescence images of each sample were taken using a fluorescence microscope (Nikon Eclipse TE2000-U). To further quantify the ROS levels, the fluorescence intensity was quantified using a multiplate reader (HH34000000, EnSight, PerkinElmer) at an excitation of 485 nm and an emission of 535 nm.

### 2.4 Bioprinting

#### 2.4.1 Cell Culture

Rat Schwann RSC96 cells were cultured in DMEM supplemented with 10% FBS, 1% penicillin-streptomycin, and 1% antibiotic-antimycotic. The cells were incubated in a humidified incubator at 37 °C with 5% CO_2_, and the culture media was changed every alternate day. The cells were passaged every 2–3 days with 0.025% trypsin-EDTA.

#### 2.4.2 Bioink Preparation

Bioink was prepared by incorporating the rat Schwann RSC96 cells into the PVA/0.75% NC gel to achieve a density of 5 × 10^6^ cells/mL. Briefly, the PVA/NC gel was sterilized under ultraviolet (UV) light for 1 h. Then, 900 μL of the PVA/NC gel was transferred to a 5 mL dispovan syringe, and a cell pellet in 100 μL of media was directly pipetted into the gel. The gel was carefully transferred to a printing cartridge, and a to-and-fro motion was used to uniformly blend the cell–PVA/NC gel suspension. The blending process was carried out gently in order to avoid the incorporation of air bubbles and to reduce damage to the cells.

#### 2.4.3 Bioprinting

A CELLINK INKREDIBLE+ extrusion-based bioprinter with a controllable pneumatic pressure was used for printing. The cell-laden primary crosslinked PVA/0.75% NC gel cartridge was attached with a nozzle having a diameter of 0.5 mm, and printing was carried at a pneumatic pressure of 15–20 psi and a printing speed of 4–6 mm/s. The bioprinted scaffold was subjected to secondary crosslinking with 1% 0.1 M NaOH for 2 min, followed by three washes with PBS. The cell culture growth media described in a previous section 2.4.1 was added to the bioprinted 3D scaffold, which was placed in a humidified incubator at 37 °C with 5% CO_2_ for 24 h.

#### 2.4.4 Cell Viability (Live/Dead) Assay

Fluorescent live/dead staining of the rat Schwann RSC96 cell-loaded 3D bioprinted scaffold was conducted to determine cell viability. Cell viability was determined at various time points, specifically at day 0 (i.e., immediately after printing), day 1, and day 7. Briefly, the 3D bioprinted scaffold was incubated for different time points in the cell culture growth media and incubated in a humidified incubator at 37 °C with 5% CO_2_. At each specific time point, the scaffold was washed three times with PBS, followed by the addition of 1 μM calcein-AM and incubation for 20 min in the dark in a humidified incubator at 37 °C. Afterward, 2 μM ethidium bromide was added to the scaffold, and it was again incubated for 10 min in a humidified incubator at 37 °C in the dark. The stained scaffold was observed and imaged using fluorescence microscopy (Nikon Eclipse TE2000-U). The images were analyzed using the ImageJ software, and the cell viability percentage was calculated.

#### 2.4.5 Cell Proliferation Assay

A cell proliferation evaluation of the rat Schwann RSC96 cell-loaded 3D bioprinted scaffold was carried out using a 3-(3,4-dimethylthiazol-2-yl)-2,5-diphenyltetrazoliium bromide (MTT) assay, following the manufacturer’s protocol. The bioprinted scaffold was printed in a standard 24-well plate, and MTT analysis was carried out at days 1, 5, and 7. At each specific time point, the cell culture media was removed and 200 μL of MTT was added, followed by 4 h of incubation in a humidified incubator at 37 °C in the dark. Afterward, the MTT solution was removed, 100 μL of DMSO was added, and the plate was placed on a shaker for 30 min in the dark to dissolve the formazan crystals. A multiplate reader (HH34000000, EnSight, PerkinElmer) was used to measure the absorbance of the solution at 570 nm, which corresponds to the number of metabolically active cells in the culture.

### 2.5 Statistical Analysis

All experiments were conducted in triplicate, and the quantitative data are represented as the average ± standard deviation. The data were analyzed using the GraphPad Prism software. The Student’s *t* test and one-way and two-way analyses of variance (ANOVA) were used as appropriate. A *p*-value less than 0.05 was considered significant.

## 3 Results and Discussion

### 3.1 Scanning Electron Microscopy and Energy Dispersive X-ray Spectroscopy

**Figure 2:**
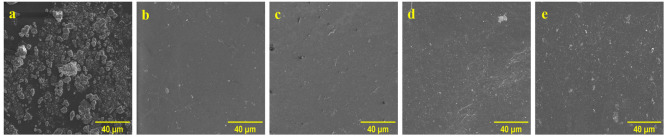
Scanning electron microscopy images at 2000× magnification of the (a) NC particles, (b) PVA/0.5% NC hydrogel, (c) PVA/0.75% NC hydrogel, (d) PVA/1% NC hydrogel, and (e) PVA/2% NC hydrogel. Scale bar: 40 μm.

The neat nanoceria (NC) particles and lyophilized PVA/NC hydrogels were imaged using a scanning electron microscope to elucidate their surface microstructures (Figure 2). It can be observed from Figure 2a that the NC particles are irregular in shape, having a varied distribution of particle size; this result is in agreement with previous reports.^50^ The presence of larger NC particles can be ascribed to the drying effect, which causes the agglomeration of the particles during the SEM sample preparation process. In the case of the lyophilized PVA/NC hydrogels, we observed a gradual increase in surface features with increasing NC loading (Figure 2b–e). This can be ascribed to the presence of NC on the hydrogel surface, which increases with increasing NC loading. We observed no pores on the surface of any of the PVA/NC hydrogels, regardless of the NC loading amount. This could be because the hydrogels fabricated for imaging were very thin.

### 3.2 X-ray Diffraction (XRD)

**Figure 3:**
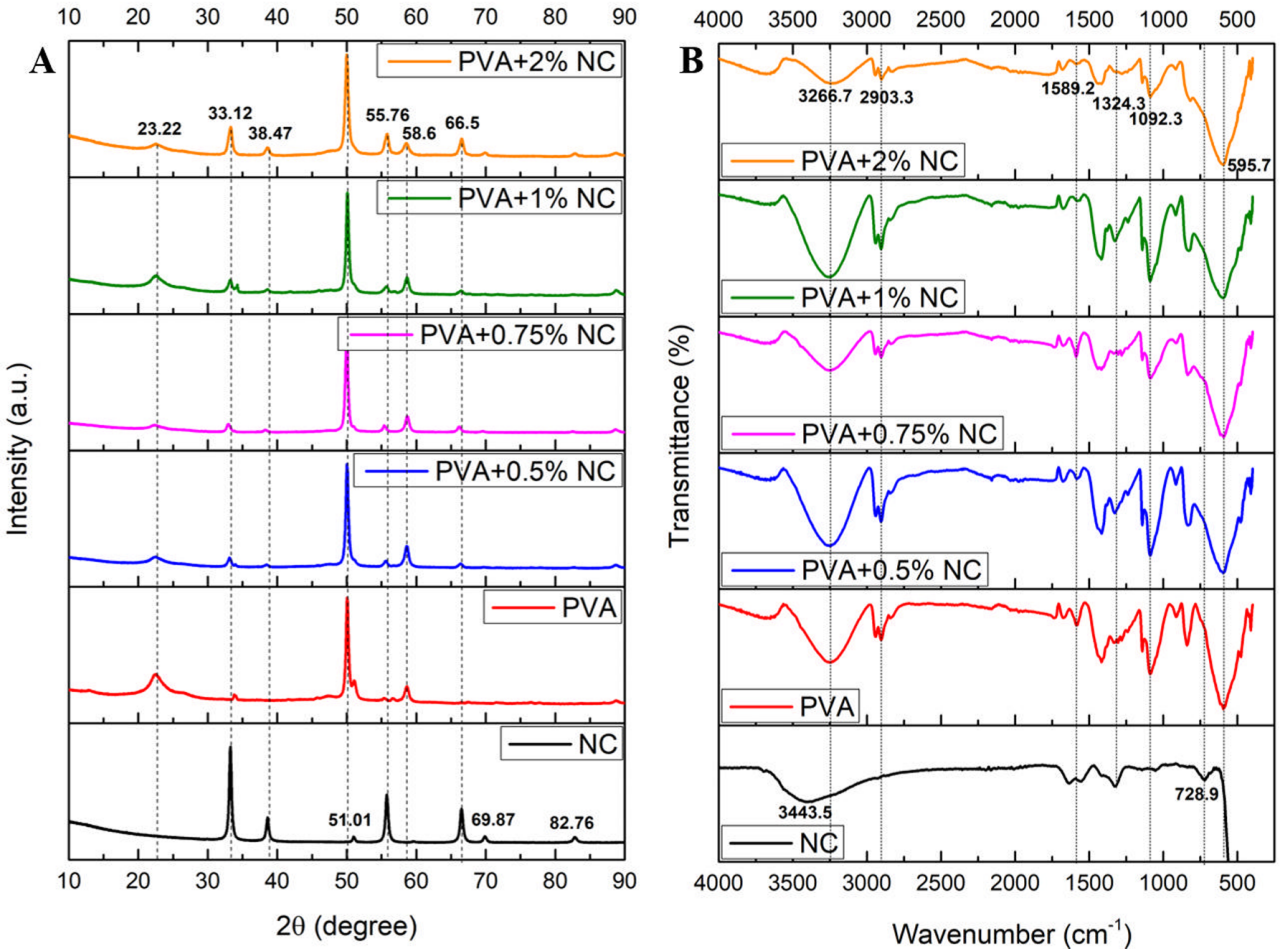
(A) XRD spectra and (B) FTIR spectra of the NC particles, neat PVA hydrogel, PVA/0.5% NC hydrogel, PVA/0.75% NC hydrogel, PVA/1% NC hydrogel, and PVA/2% NC hydrogel.

The XRD spectra of the NC particles shows characteristic peaks at 2θ = 33.12°, 55.76°, 69.87°, and 82.7°, which can be indexed as the (200), (311), (400), and (420) planes, respectively (Figure 3A); these results are in agreement with previously published reports.^51,52^ In the case of the neat PVA hydrogel, a broad peak between 2θ = 20–25° is assigned to the polymer matrix. The broadness of the peak indicates the amorphous nature of the polymer, which is in agreement with previously published reports.^53−56^ In the case of the PVA/NC hydrogels, we observed characteristic peaks for PVA and NC at 2θ = 20–25° (broad peak), ∼33.12°, and 55.76°, which were assigned to PVA and the (200) and (311) planes of NC, respectively. We also observed an expected increase in the intensity of the characteristic NC peaks as the NC loading increased for the PVA/NC hydrogels (Figure 3A). The sharp peaks at 2θ = ∼50° and ∼58° that are observed for the neat PVA and PVA/NC hydrogels are ascribed to the metal sample holder used for the XRD analysis.

### 3.3 Fourier Transform Infrared (FTIR) Spectroscopy

The structural determination of the nanocomposite was completed using FTIR spectroscopy. The band at ∼728 cm^–1^ is characteristic of Ce–O stretching vibrations,^57^ while the broad band ∼3443 cm^–1^ corresponds to O–H stretching vibrations, which can be attributed to surface-adsorbed water molecules. For the neat PVA hydrogel, we observed prominent bands at 820, 1092, ∼1450, 2903, and 3266 cm^–1^, which can be assigned to C–C stretching, C–O stretching, C–H deformation, CH_2_ stretching, and O–H stretching vibrations, respectively. We observed the combination of characteristic NC and PVA bands in the composite PVA/NC hydrogels, thus confirming the presence of both components (Figure 3B).

### 3.4 Dynamic Light Scattering (DLS) and Zeta Potential Analyses

**Figure 4:**
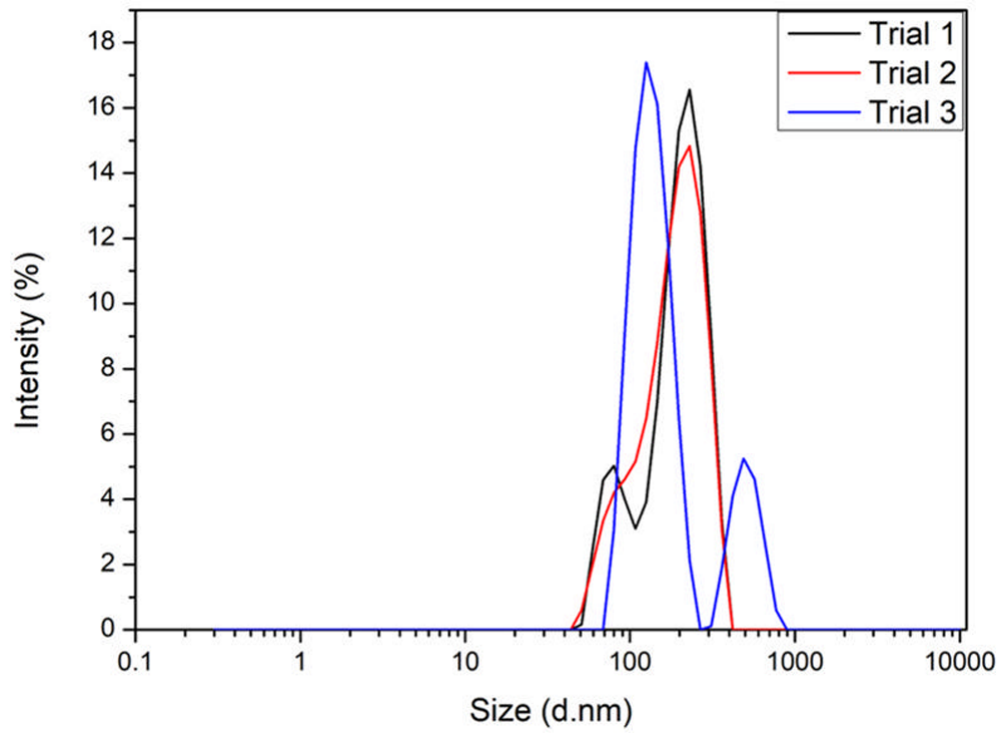
Dynamic light scattering analysis of the NC particles.

DLS analysis was conducted to determine the hydrodynamic radius of the NC particles when in an aqueous dispersion. The hydrodynamic radius of the neat NC particles was determined to be ∼410 nm (Figure 4). The multimodal profile and high PDI of 0.42 indicate a wide distribution of particle sizes and, potentially, an extent of particle agglomeration. The particle size distribution obtained by DLS coincides with the size variability observed under SEM. The zeta potential analysis revealed a surface charge of +27.36 mV, which is indicative of the cationic nature of these particles.

### 3.5 UV–Vis Spectroscopy

**Figure 5:**
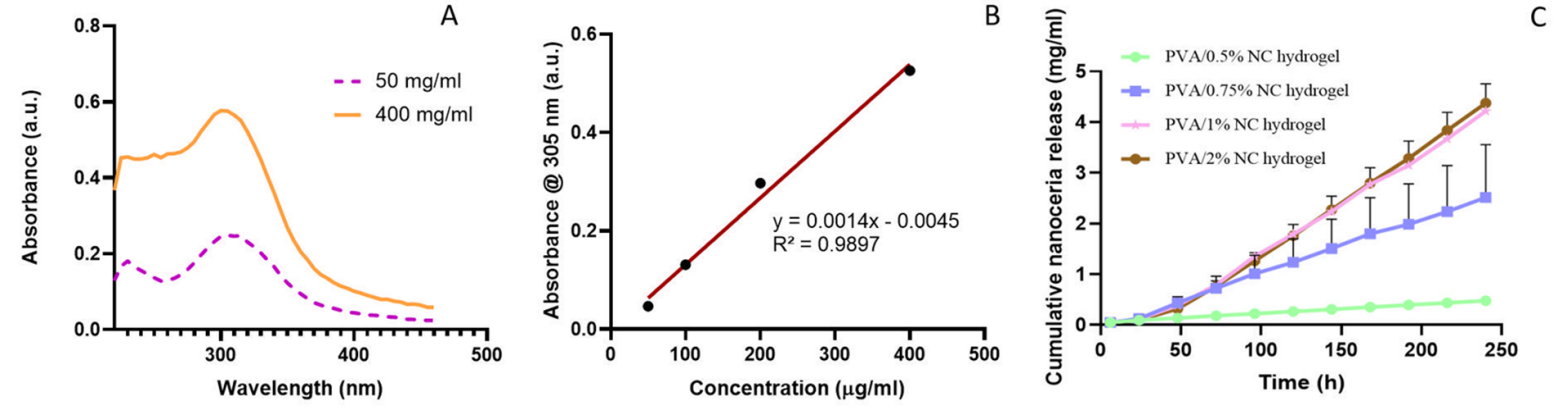
(A) UV–Vis spectra of nanoceria at 50 and 400 mg/mL. (B) Standard curve of nanoceria (the error bars are too small relative to the average values to be clearly visible). (C) Cumulative release of nanoceria from all concentrations of the PVA/NC hydrogels in PBS at various time points at 37 °C. Data are presented as the average ± standard deviation (*n* = 3).

To study the release of NC from the hydrogels, we conducted UV–Vis analysis. We first conducted UV–Vis analysis on the aqueous dispersion of NC at two different concentrations (50 and 400 mg/mL). We observed a characteristic NC peak at 305 nm, the intensity of which increased with an increasing concentration of NC (Figure 5A). Then, we conducted NC release from the different lyophilized PVA/NC hydrogels in PBS over time. To analyze the nanoceria release quantitatively, the concentration of the released nanoceria was calculated using the calibration curve (Figure 5B). As shown in Figure 5C, all of the hydrogels exhibited a gradual release of NC over a period of 240 h (10 days). Interestingly and as anticipated, we observed the highest NC release for the PVA/2% NC hydrogel, which reached a maximum release of ∼4 mg/mL by day 10; the lowest NC release was observed for the PVA/0.5% NC hydrogel, which reached a maximum release of ∼0.5 mg/mL by day 10. The trend of NC release was PVA/2% NC > PVA/1% NC > PVA/0.75% NC > PVA/0.5% NC.

### 3.6 Free Radical Scavenging Activity

The antioxidant properties of the lyophilized PVA/NC hydrogels were investigated using a 2,2-diphenyl-1-picrylhydrazyl (DPPH) assay. The assay functions when DPPH radicals get neutralized upon receiving electrons or hydrogen atoms; this results in the solution changing color from purple to yellow. The intensity of the yellow color depends on the amount of neutralization of the DPPH radicals, which can be quantified by measuring the absorption at 517 nm.^47^ It was observed that the inclusion of NC within the lyophilized PVA hydrogels caused a significant increase (*p* < 0.0001) in the antioxidant activity when compared to the neat PVA hydrogel, regardless of the NC concentration (Figure 7A). Furthermore, among the lyophilized PVA/NC hydrogels with differing NC concentrations, we observed no significant difference in antioxidant activity, although a marginal reduction in antioxidant activity was observed at the highest loading of NC (the PVA/2% NC hydrogel) when compared to the other PVA/NC hydrogels. The antioxidant activity observed in all concentrations of the lyophilized PVA/NC hydrogels is ascribed to the release of NC from the hydrogels. This observation is in line with previously published reports, where it was determined that the concentration of NC in a hydrogel was directly proportional to the free radical scavenging activity of the hydrogel.^45,47^ In addition, we also observed some antioxidant activity in the neat PVA hydrogel control. This can be attributed to the presence of citric acid, which was incorporated as a primary crosslinker during the fabrication of the hydrogel. The free radical scavenging activity of citric acid has been reported previously when it was used as a crosslinker to prepare chitosan hydrogels.^58,59^ Therefore, we are able to conclude that our dual crosslinked PVA/NC hydrogels had better antioxidant activity than the neat PVA hydrogel.

**Figure 6:**
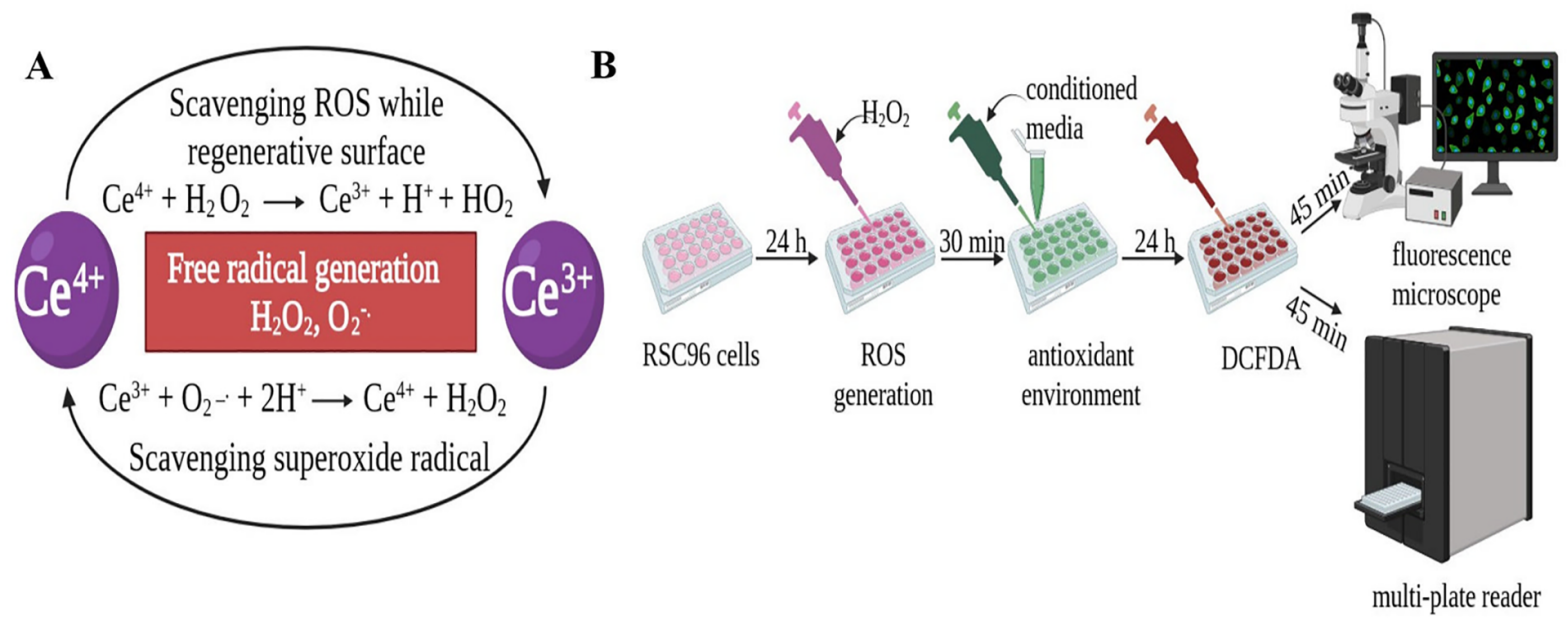
Schematics representing (A) the antioxidant mechanism of nanoceria and (B) the protocol for the H_2_DCFDA assay.

**Figure 7:**
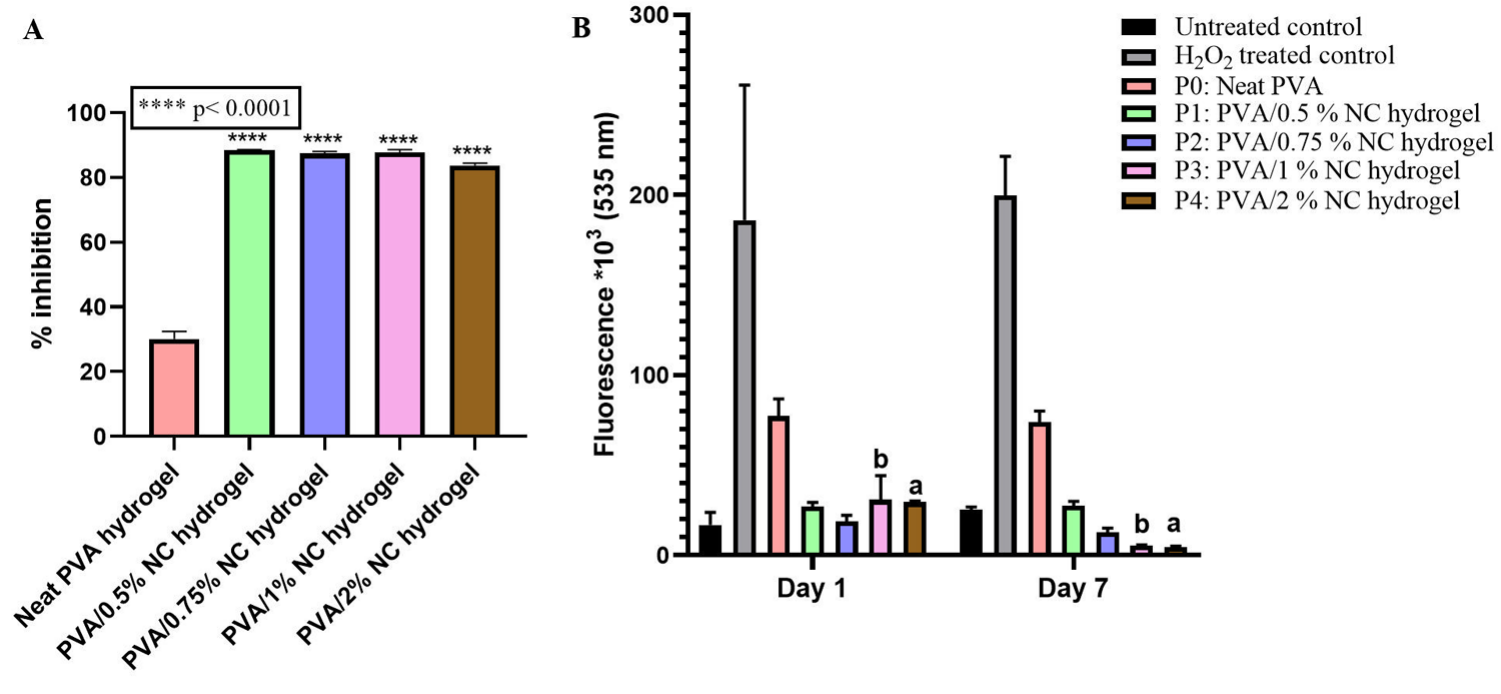
(A) DPPH assay of the PVA/NC hydrogels with different concentrations of NC. Statistical analysis was done using the two-way analysis of variance (ANOVA) and Tukey’s test with ****p < 0.0001. Data are presented as the average ± standard deviation (*n* = 3). (B) H_2_DCFDA assay of the PVA/NC hydrogels with different NC loading amounts. Untreated cells and H_2_O_2_-treated cells are used as controls. Statistical analysis was done using the Student’s *t* test; in the graph, a represents *p* < 0.0001 and b represents *p* < 0.05. Data are presented as the average ± standard deviation (*n* = 3).

**Figure 8:**
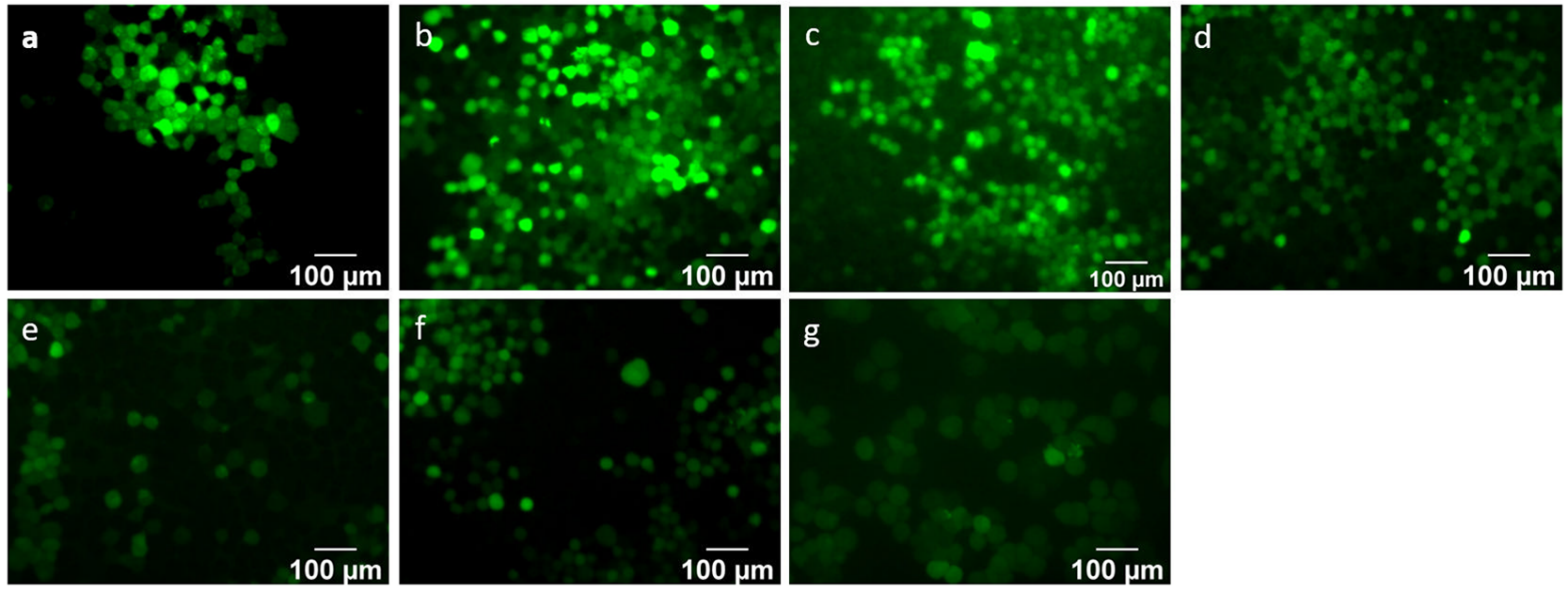
Representative fluorescence microscopy images of the intracellular levels of ROS in the Schwann cells treated with the extract from the scaffold immersed in complete media for 7 days. After a 24 h treatment, the cells were stained with 2′,7′-dichlorodihydrofluorescein diacetate (H_2_DCFDA) and observed with a fluorescence microscope: the (a) untreated control, (b) H_2_O_2_-treated control, (c) neat PVA hydrogel, (d) PVA/0.5% NC hydrogel, (e) PVA/0.75% NC hydrogel, (f) PVA/1% NC hydrogel, and (g) PVA/2% NC hydrogel. Scale bar: 100 μm.

Next, we conducted an in vitro H_2_DCFDA assay to corroborate the findings from the DPPH assay. The difference between the two assays was that the H_2_DCFDA assay was performed in the presence of cells, unlike the DPPH assay, which was performed in PBS. The H_2_DCFDA assay was employed to investigate the intracellular free radical scavenging (antioxidant) activity in the rat Schwann RSC96 cells using fluorescence microscopy and spectrofluorometry. H_2_DCFDA is highly cell-permeable and acts as an intracellular probe in the presence of free radicals. H_2_DCFDA is cleaved at the ester bonds by the esterase present within the cell, resulting in the production of the cell-impermeable compound H_2_DCF. When exposed to free radicals, H_2_DCF is reduced to the highly fluorescent DCF molecule, the intensity of which is measured at 530 nm, which corresponds to the amount of free radicals.^48^ In this experiment, we used untreated cells, H_2_O_2_-treated cells, and the neat PVA hydrogel as controls. H_2_O_2_ was used to induce ROS generation in the cells. The cells were cultured on a 24-well plate and were stimulated with H_2_O_2_ to study the antioxidant response of the conditioned media containing NC that was released from the different PVA/NC lyophilized hydrogels (Figure 6B). The fluorescence microscopy images in Figure 8 show reduced fluorescence intensity in the PVA/NC hydrogels with higher NC concentrations, indicating that the free radical scavenging activity increases with an increasing concentration of NC in the PVA/NC hydrogels. We maintained the microscopy settings between the different samples and also seeded the same number of cells in each condition to ensure that the observed differences in the fluorescence intensity are indeed due to the antioxidant effect of NC.

Next, we quantified the free radical scavenging activity by analyzing the fluorescence intensity measured by fluorescence spectroscopy (Figure 7B). From the quantitative analysis, we observed no noticeable change in the fluorescence intensity of the untreated cells over a period of 7 days. In the case of the H_2_O_2_-treated cells, an expected increase in the fluorescence intensity was observed at both days 1 and 7, which is indicative of a significant amount of ROS in the culture. We also observed an increase in the fluorescence intensity in the neat PVA hydrogel control compared to the untreated cells at both days 1 and 7. However, the extent of the increase was significantly smaller than the increase observed in the H_2_O_2_-treated cells. Taken together, the neat PVA hydrogel appears to inhibit the ROS production associated with the H_2_O_2_ treatment by exhibiting some antioxidant response, which we ascribe to the presence of citric acid (which was used as primary crosslinker in the PVA hydrogel). We suspect the antioxidant property displayed by the neat PVA is due to the presence of citric acid. It has been previously reported that citric acid can neutralize ROS by the direct transfer of hydrogen atoms.^58−61^ In the case of the PVA/NC hydrogels, we observed significantly lower fluorescence intensities in all of the PVA/NC hydrogels compared to the neat PVA hydrogel control and the H_2_O_2_-treated cell control at both days 1 and 7. The observed reduction in the fluorescence intensity is proposed to have been caused by the combination of (i) the presence of citric acid in the hydrogels and (ii) the release of NC from the hydrogels. Studies have shown that NC has an inherent property to modify into two oxidative states, i.e., Ce^3+^ and Ce^4+^ (Figure 6A), thus providing oxygen vacancies for free radical scavenging; this enables NC to mimic the biological activity of antioxidant enzymes, such as superoxide dismutase and catalase.^46,62−64^ No significant change in fluorescence intensity was observed between days 1 and 7 in the case of the PVA/0.5% NC and PVA/0.75% NC hydrogels. In contrast, the fluorescence intensity reduced significantly between days 1 and 7 in the case of the PVA/1% NC (*p* < 0.05) and PVA/2% NC (*p* < 0.0001) hydrogels.

### 3.7 Bioprinting

First, the bioink composition and crosslinking conditions were systematically analyzed. We employed our dual crosslinking strategy for printing the PVA/NC bioink. The PVA/NC gel was first crosslinked with citric acid during the gel preparation stage. First, an initial optimization process involving various concentrations of PVA was carried out. At a concentration of 25 wt % PVA, the gel exhibited non-flow behavior, indicating its initial bulk resting state (Figure S1). Printing with the 25 wt % PVA gel was carried out, and it was observed that, even with the smallest nozzle size (27G) and the lowest pressure (0.72 psi), the gel was extruded without achieving proper printing. The subsequent step involved optimizing two concentrations of the primary crosslinker, citric acid (1 and 5 wt %), for the printing parameters. After completing initial printing tests, it was observed that the PVA gel with 1 wt % citric acid exhibited low resolution, and the formulation was unable to yield a proper print (Figure S2 and Video S1). Next, the printing parameters for the PVA gel with 5 wt % citric acid were optimized. We observed that, when the nozzle sizes 20G and 22G were used, a grid structure could not be accurately printed. However, when a 25G nozzle and a pressure of 19 psi were used, a uniform print was obtained (Figure S2 and Video S2). A 27G nozzle was also used to analyze printability. Although using a smaller nozzle size typically improves printability, we observed that at a nozzle size of 27G, nozzle clogging occurred and printing could not be carried out. Furthermore, it was observed that, when PVA and citric acid were used in a 5:1 wt % ratio, proper gelation occurred regardless of the NC concentration, and a uniform flow of the filament was obtained during printing (Figure S2). It can be suggested that when PVA and NC are combined, NC becomes incorporated between the polymer chains. Furthermore, when citric acid is incorporated into the PVA/NC gel, esterification reactions can take place between the hydroxyl groups of PVA and the carboxyl groups in citric acid, forming inter- and intramolecular ester linkages in the polymer. These reactions cause a reduction in the dissolution of the scaffold in water after printing, as they reinforce the intramolecular binding by providing covalent bonds, in addition to hydrogen bonds.^61,65^ Primary crosslinking provided a stable viscosity to the bioink, enabling it to print successfully, and a printed grid structure could be obtained. However, this printed structure lacked stability and shape fidelity. We observed that the printed scaffold easily dissolved in water over a period of 24 h. We also observed that increasing the concentration of citric acid caused a rapid increase in the viscosity, making it difficult to extrude the PVA/NC gel through the nozzle during printing. Therefore, we hypothesized about performing secondary crosslinking on the printed scaffold, which would be carried out after the printing process. For this purpose, we selected NaOH for its ionic crosslinking abilities and used it to impart stability in the natural polymer hydrogels. We tried different concentrations of NaOH and varying crosslinking times to obtain a stable, robust scaffold. We observed that the scaffold was able to maintain its integrity when immersed in a 1% w/v 0.1 M NaOH solution for 2 min. The addition of NaOH has been reported to induce a level of phase separation of the polymer network, thereby increasing the microcrystallinity of semicrystalline polymers such as PVA.^66,67^ Once the crosslinking conditions were finalized, we optimized the number of cells to be included within the bioink.

**Figure 9:**
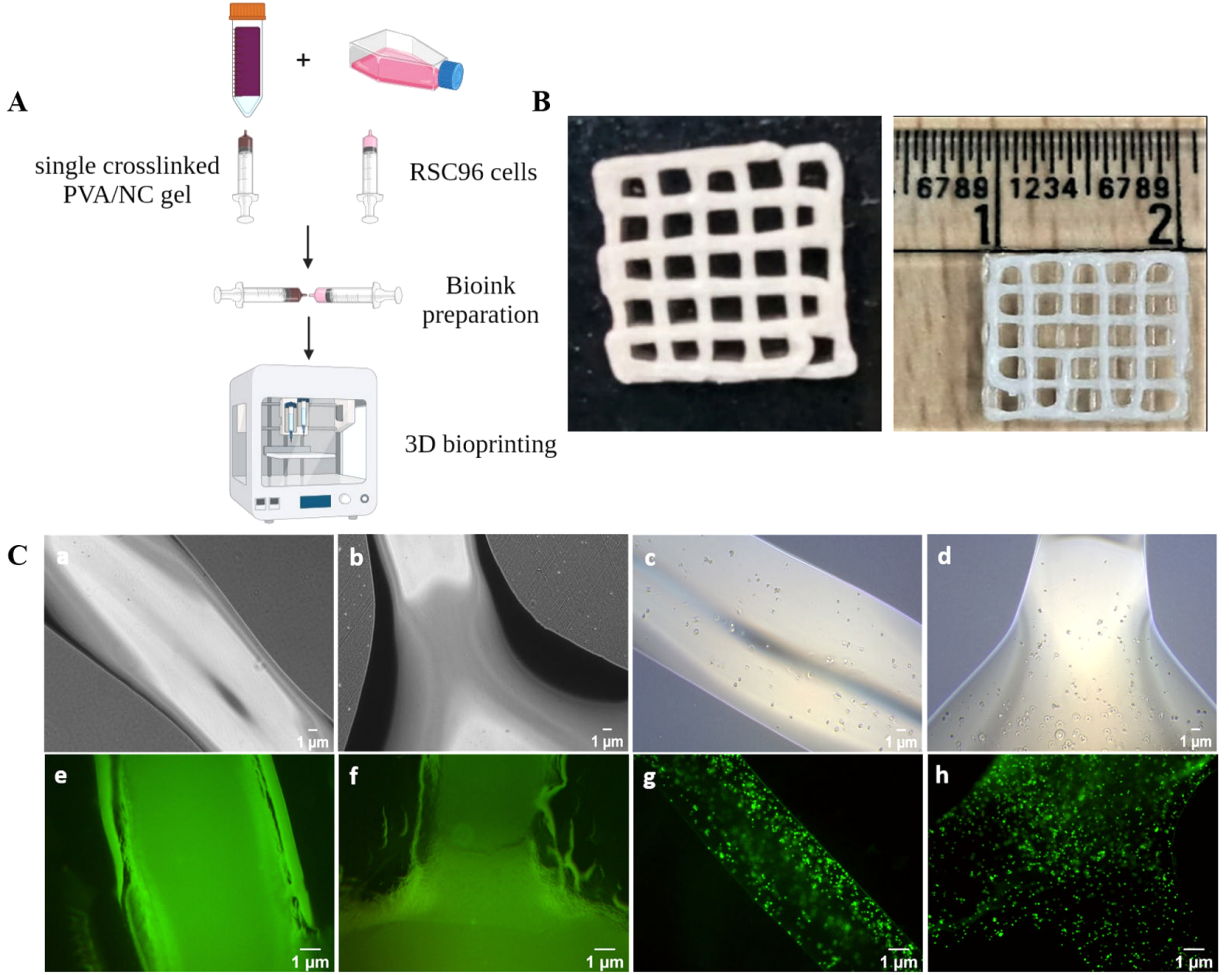
(A) Schematic showing the manipulation of the PVA/0.75 wt % NC gel and the RSC96 cells to obtain bioink for 3D bioprinting. (B) Representative images of the 3D bioprinted scaffold with dimensions of 10 × 10 × 1.2 mm. (C) Representative phase contrast microscopy images of the printed scaffold (a, b) without cells and (c, d) laden with cells immediately after printing. Representative fluorescence microscopy images of the live/dead assay of the printed scaffold (e, f) without cells and (g, h) laden with cells 24 h post-bioprinting. The round structures observed in the printed scaffold represent rat Schwann RSC96 cells (all time points are shown in Figure S4). Scale bars: 1 μm.

The bioink was prepared using rat Schwann RSC96 cells, as they are the primary glial cells in the peripheral nervous system and are known to promote axonal regeneration.^68,69^ Rat Schwann RSC96 cells provide growth pathways by rapidly changing their phenotype, leading to myelin formation around axons after any peripheral nerve injury.^70^ In our bioink, 5 × 10^6^ cells were taken in 100 μL of media in one syringe, and 900 μL of the PVA/0.75% NC gel was loaded into another syringe to achieve the total cell loading (5 × 10^6^ cells/mL). Both syringes were connected via a female–female luer lock adapter, and the gel and cells were mixed thoroughly using a to-and-fro motion with minimal pressure application. After thorough mixing, the bioink was loaded into a printing cartridge for bioprinting (Figure 9A).

A 3D bioprinted scaffold of the PVA/0.75% NC gel was fabricated with a CELLINK INKREDIBLE+ bioprinter using a CAD model of a rectangular grid structure (10 × 10 × 1.2 mm). A rectangular grid-like pattern was chosen for bioprinting, as the structure has regularly spaced pores. These pores facilitate a robust supply of the media, nutrients, and oxygen to the cells. The extrusion process was carried out at 25 °C, and a grid-shaped structure was printed at a feed rate of 600 mm/min with a line spacing of 2 mm. Printing was optimized using various nozzle sizes and different pressures, as described above (also see Table S1). Printing conducted with a 25G nozzle and 19 psi of pressure yielded smooth uniform filaments for fabricating the grid structure (Figure S2). The 3D structure printed with a 25G nozzle exhibited smooth, uniform filaments and was selected as an ideal scaffold for maintaining the integrity of the printed construct and providing high cell viability, as has been suggested in previous studies.^12,71,72^ To maintain shape fidelity, the cell-laden bioprinted scaffold was subjected to a second crosslinking step by completely immersing it in a 1% w/v 0.1 M sodium hydroxide solution for 2 min.

To analyze whether our PVA/NC bioink and dual crosslinking strategy were compatible with cells and whether the pressure applied during bioprinting had any harmful effect on the cells, a cell viability study was carried out.

### 3.8 Cell Viability in the Printed Scaffold

Images a and b in Figure 9C are representative magnified phase contrast images of the 3D bioprinted scaffold without cells, and images e and f in Figure 9C are representative magnified fluorescence images of the calcein-AM/ethidium bromide-stained bioprinted scaffold without cells. Images c and d in Figure 9C are representative magnified phase contrast images of the rat Schwann RSC96 cell-laden 3D bioprinted scaffold immediately after printing, and images g and h in Figure 9C are representative magnified fluorescence images of the calcein-AM/ethidium bromide-stained, cell-laden bioprinted scaffold after a 24 h culture. It can be seen that the 3D bioprinted scaffold retained its shape during the 24 h culture when comparing the fluorescence images to the phase contract images. In the phase contrast images that were taken immediately after printing, cells with a more rounded morphology are clearly visible and are observed to be encapsulated within the bioprinted scaffold (Figure 9C, images c and d). From the fluorescence images (Figure 9C, images g and h), the presence of a predominantly green fluorescence with only negligible red stains of dead cells indicates that (i) the cells are homogeneously dispersed within the printed scaffold (which is indirectly indicative of a uniform cell dispersion in the bioink), and (ii) cell viability was maintained during both the printing and crosslinking processes, as well as during encapsulation for 24 h. Furthermore, some extent of the cells having an elongated cell morphology was observed on day 7 (Figure S4). The cell viability was calculated, and when comparing day 0 and day 1, no significant reduction in the cell viability percentage was observed. While a slight reduction in the cell viability percentage was observed on day 7, it was not significant, which suggests that the bioink processing and printing conditions exerted no adverse impact on the encapsulated cells. From the cell viability data (Figure S4), we could also observe that our PVA/NC blend, along with the dual crosslinking strategy, was not toxic to the RSC96 cells.

Cell proliferation was further analyzed using an MTT assay. We observed that there was a gradual increase in cell proliferation from day 1 to day 7 in the neat PVA and PVA/2% NC hydrogels. Cell proliferation also increased gradually from day 1 to day 5 in the PVA/0.5% NC, PVA/0.75% NC, and PVA/1% NC hydrogels, and there was a notable (though not a significant) reduction from day 5 to day 7 in these hydrogels (Figure S5). With both these cell viability and cell proliferation results, we observed that the RSC96 cells were able to withstand the shear forces that were induced during the printing process and that the shape fidelity and integrity of the 3D bioprinted scaffold were maintained. We believe that the combination of the biocompatibility of the scaffold and the antioxidant properties of NC can be used to promote axonal regeneration after peripheral nerve injury. The above results show that our PVA/NC composite, along with the dual crosslinking strategy, can be used as a bioink for treating peripheral nerve injury.

## 4 Conclusion

In this study, a 3D bioprinted nanocomposite scaffold was fabricated using a PVA/NC blend and a dual crosslinking strategy. The incorporation of NC in the PVA/NC gels was confirmed through various analyses. To enhance the printability and shape fidelity of the bioprinted scaffold, a dual crosslinking strategy was employed. This dual crosslinking process involved the addition of citric acid as a primary crosslinker to the PVA/NC gel, which helped to obtain a viscous gel that could be used for bioprinting. The subsequent addition of sodium hydroxide as a secondary crosslinker post-bioprinting helped the printed structure maintain its shape. It was also demonstrated that, when cells were incorporated into the PVA/NC gel to obtain a bioink, cell viability was maintained even under the shear forces induced during the printing process. Additionally, the PVA/NC hydrogels exhibited free radical scavenging properties, which are crucial for reducing the oxidative stress at peripheral nerve injury sites. It was observed that there was an increase in the free radical scavenging activity as the concentration of NC in the PVA/NC hydrogels increased. Overall, the combination of PVA/NC gel and the dual crosslinking strategy that employs citric acid and sodium hydroxide could be a viable 3D bioprinting method for preparing scaffolds that can treat peripheral nerve injury.

## Supporting Information Available

Optimization of the concentration of PVA, optimization of the amount of primary crosslinker (citric acid), optimization of the printing parameters (nozzle size and pressure), images of the scaffold after incubation at different times, fluorescence microscopy images of the scaffold at different times post-bioprinting, cell viability study results, and cell proliferation assay results (PDF)
Video S1: bioprinting using the PVA gel with 1 wt % citric acid (MP4)
Video S2: bioprinting using the PVA gel with 5 wt % citric acid and employing a 25G nozzle and 19 psi of pressure (MP4)
