# Supplementary material for: Dual Crosslinked Antioxidant Mixture of Poly(vinyl alcohol) and Cerium Oxide Nanoparticles as a Bioink for 3D Bioprinting
Source: ACS Appl Nano Mater. 2023 Sep 25;7(16):18177–88. doi: 10.1021/acsanm.3c02962 (PMC11348314; doi:10.1021/acsanm.3c02962)
Supplement: Supplementary file 1 — an3c02962_si_001.pdf [file an3c02962_si_001.pdf]

Dual Crosslinked Antioxidant Mixture of Polyvinyl Alcohol and Cerium Oxide Nanoparticles as a Bioink for 3D Bioprinting

Nasera Rizwana,[1] Namrata Maslekar,[2] Kaushik Chatterjee,[3] Yin Yao,[4] Vipul Agarwal,[2*] Manasa Nune[1*]

[1] Manipal Institute of Regenerative Medicine (MIRM), Bengaluru, Manipal Academy of Higher Education (MAHE), Manipal 576104, Karnataka, India
[2] Cluster for Advanced Macromolecular Design (CAMD), School of Chemical Engineering, University of New South Wales, Sydney, NSW 2052, Australia
[3] Department of Materials Engineering, Indian Institute of Science, Bangalore, 560012 India.
[4] Electron Microscope Unit, Mark Wainwright Analytical Centre, University of New South Wales, Sydney, New South Wales 2052, Australia

* Corresponding authors: agarwalvipul84@gmail.com; manasa.nune@manipal.edu

Supporting Information

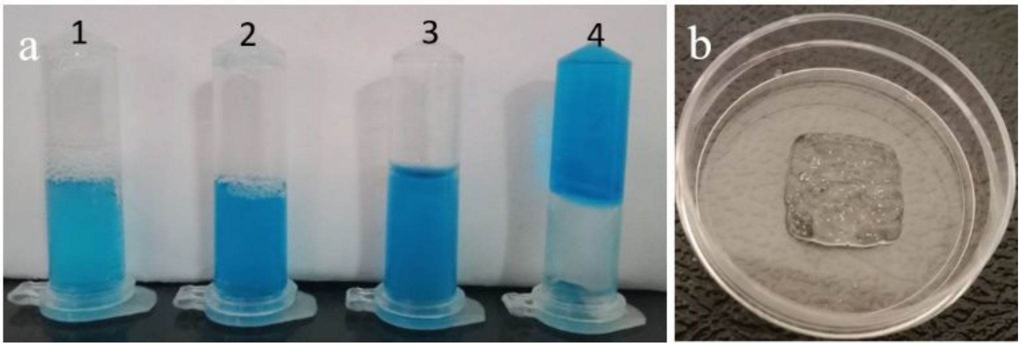

Figure S1: Optimization of concentration of PVA. a) Non-flow behavior of various concentrations of PVA. Eppendorf tubes 1- 5 wt% PVA, 2- 10 wt% PVA, 3- 20 wt% PVA and 4- 25 wt% PVA. Blue color was added to obtain better clarity images; and b) 3D printed scaffold prepared using 25 wt% PVA printed with 27 G nozzle and 0.72 psi pressure resulting in an improper print.

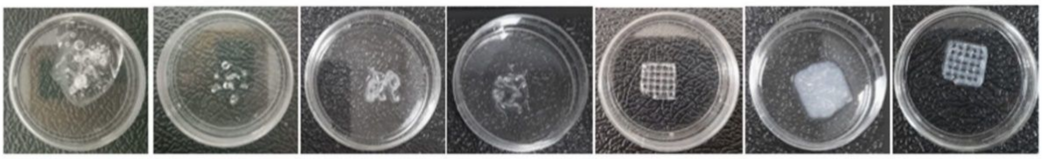

PVA (wt%)	25	25	25	25	25	25	25
Citric acid (wt%)	1	1	5	5	5	5	5
NC (wt%)	0	0	0	0	0	0.75	0.75
Nozzle size (G)	25	27	20	22	25	22	25
Printing pressure (psi)	3.4	35.67	8.9	14.5	19	2.9	19

Figure S2: Optimization of primary crosslinking using citric acid. Different concentrations of citric acid were mixed to the 25 wt% of PVA solution prior to printing and resulting printed matrices are presented. It was observed that with the increase in citric acid concentration, better printing was obtained. At 5 wt% of citric acid, 25 G nozzle size, and 19 psi pressure a proper print was obtained.

Table S1: Table representing optimization of nozzle size and printing pressure used for bioprinting PVA/0.75% NC solution crosslinked with 5 wt% citric acid. The optimum print was obtained with a nozzle size of 25 G at 19 psi pressure. × indicates improper print obtained, and ● indicates proper print obtained.

Nozzle size (G)	Pressure (psi)	Printing status
20	07	×
22	11	×
25	19	●
27	1- 43	×

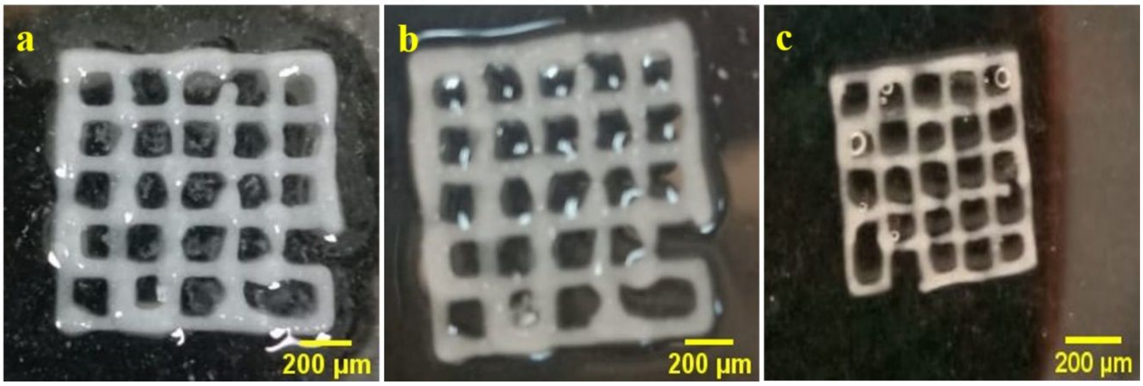

Figure S3: Representative images of a printed scaffold after incubation at ℃ in an in at a) day 0, b) day 1, and c) day 7. Scale bar = 200 µm.

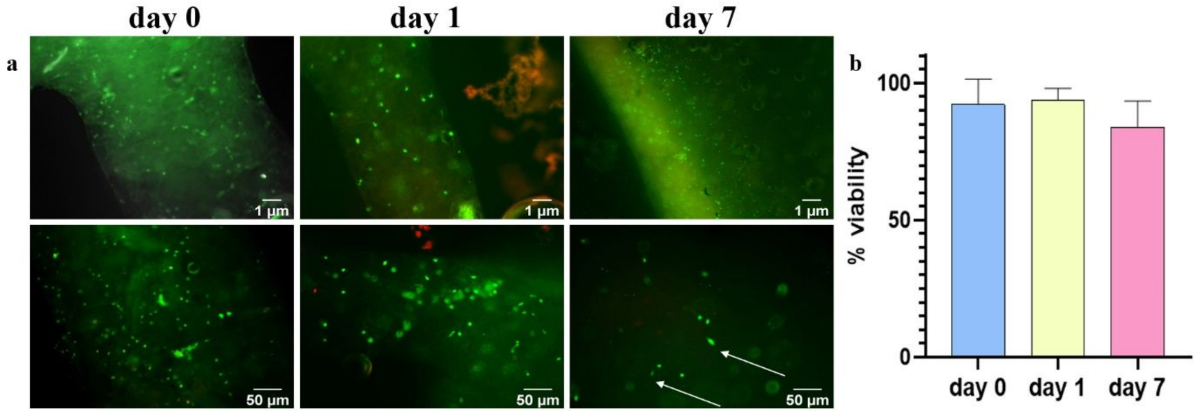

Figure S4: a) Representative fluorescence images of live/dead assay of the printed scaffold with cells at day 0, day 1 and day 7 post-printing. The top panel represents images at 4X magnification, and the bottom panel represents images at 20x magnification. The viable RSC96 cells can be seen as green. Some cells with elongated morphology were also observed on day 7 (white arrows). Scale bars: top panel = 1 µm and bottom panel = 50 µm. b) A plot representing percentage viability of RSC96 cells at different time points within the bioprinted scaffolds. We observed >95% viability of encapsulated cells in printed scaffolds over 7 days. Statistical analysis was conducted using a one-way ANNOVA with Tukey *post hoc* test. Data are presented as average ± standard deviation (n=5).

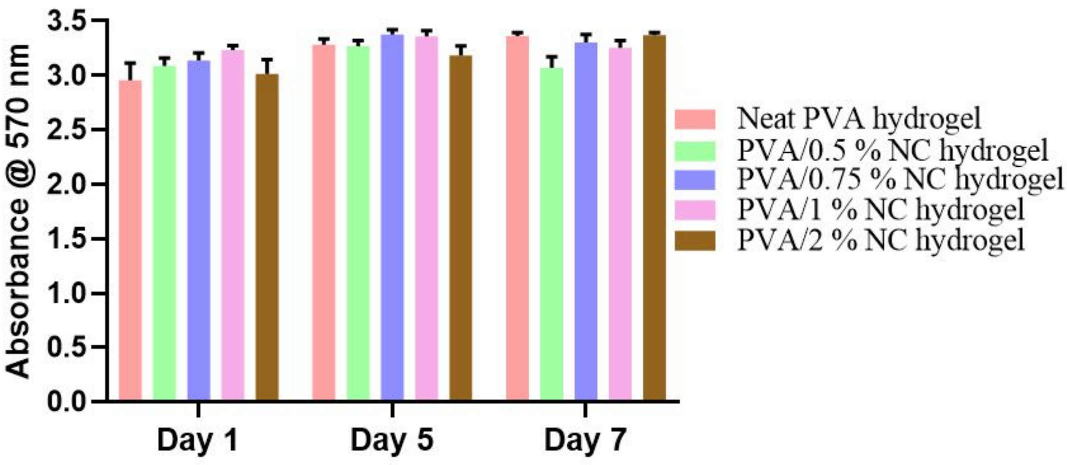

Figure S5: Cell proliferation assay showing cell growth over a period of 7 days post incubation. Statistical analysis was done using two-way ANNOVA with Bonferroni *post hoc* test. Data are presented as average ± standard deviation (n=9).
